# Supplementary material for: Coronary calcium scoring assessed on native screening chest CT imaging as predictor for outcome in COVID-19: An analysis of a hospitalized German cohort
Source: PLoS One. 2020 Dec 30;15(12):e0244707. doi: 10.1371/journal.pone.0244707 (PMC7773182; doi:10.1371/journal.pone.0244707)
Supplement: Attachment — Submitted filename: Rebuttal Letter CAC in COVID-19 Plos One FINAL.docx [file pone.0244707.s002.docx]

**Submitting Original Article to PLOS ONE**

Title: “**Coronary Calcium Scoring assessed on native screening chest CT imaging as predictor for outcome in COVID-19: an analysis of a hospitalized German cohort”**

Authors:

Gregor S. Zimmermann, Alexander Fingerle, Christina Müller-Leisse, Felix Gassert, Claudio E. von Schacky, Tareq Ibrahim, Karl-Ludwig Laugwitz, Fabian Geisler, Christoph Spinner, Bernhard Haller, Markus R. Makowski and Jonathan Nadjiri

Dear Editor,

Enclosed you will find the revised version of our manuscript “Coronary Calcium Scoring assessed on native screening chest CT imaging as predictor for outcome in COVID-19: an analysis of a hospitalized German cohort”, which we would like to resubmit to PLOS ONE.

We have addressed each of the journal requirements, the editors ‘comments and the review comments to the author. By responding to the detailed and thoughtful comments of the reviewer, the paper has been substantially improved. Changes in the manuscript are marked.

Thank you very much for reconsidering our manuscript for publication in PLOS ONE.

With best regards,

Gregor S. Zimmermann, M.D.

Department of Internal Medicine I, Division of Respiratory Diseases

Klinikum rechts der Isar, Technical University Munich

Ismaninger Str. 22

81675 Munich, Germany

gregor.zimmermann@tum.de

We thank the reviewers for your helpful comments. By addressing your suggestions, the manuscript has been significantly improved. We marked changes in the text. The comments have been addressed as follows:

Journal Requirements:

1. We have checked whether the manuscript meets the requirements of PLOS ONE.
2. We provided additional information about the patient’s records reviewed for our retrospective study. The IRB waived the requirements for informed consent and we added this important information to the manuscript. Data acquisition and processing has been approved for this particular study by the local ethics committee.
3. We corrected the title as suggested.
4. a) Following local laws and Clinical Institutional Review Board the data sharing is restricted due to potentially identifying patient information in this study. We added a excel sheet of the CAC and the endpoints of our study. An identification of an individual patient is not possible in this excel sheet. A reasonable request for a data access could be sent by researchers who meet the criteria for access to confidential data to the corresponding author only after permission from the Clinical Institutional Review Board (Ethic Committee of the Technical University Munich, Director: Professor Dr. G. Schmidt, contact: [ethikkommission@mri.tum.de](mailto:ethikkommission@mri.tum.de)).

b) We uploaded a minimal anonymized data set as supporting information file.

5. Tables were included as part of the manuscript and the individual files were removed.
6. We corrected this and included our ethics statement in the methods section and deleted it from another section.

Answer to Additional Editors Comments:

The editors raised an interesting question of any correlation with inflammatory markers. From the perspective of a tertiary medical centre many patients were transferred from external hospitals after several days for intensive care treatment, so comparable time points of inflammatory markers couldn´t be established. However, the editor added a valuable comment and we included this point in the discussion as an important goal of further investigation.

Review Comments to the Author:

1) We added a more detailed description of individual chart review in the methods section. Due to transfer of sedated and intubated patients from external hospitals to our tertiary medical centre a complete data set of the medical history was not obtainable in all cases. The main purpose of our study was to identify a parameter to predict the individual patients‘ risk with COVID-19 independently from clinical information and medical history by a CT scan on admission.

2) We added more precise information regarding ICU admission reasons in the method´s section. Our hospital has established a clearly defined protocol for admission on ICU. Admission to ICU was indicated by the treating physicians in case of poor oxygenation despite supplemental oxygen or secondary organ failure. According to our hospital COVID-19 protocol poor oxygenation was defined as respiratory rate> 30/min and oxygen saturation <90% despite 8 liter/minute supplemental oxygen via mask. Due to compensated ICU capacities there was no delay in admission to ICU or indication for triage. We added this information in the methods section.

Thank you very much for receiving our manuscript and considering it for review. The editors and reviewers comments have overall augmented the value of our paper. We hope that our manuscript meets your high scientific standards and will be eligible for publication in *PLOS ONE.* We appreciate your time and look forward to your response.

Sincerely,

Gregor Zimmermann, M.D.

Department of Internal Medicine I

Klinikum rechts der Isar

Technical University Munich

Ismaninger Str. 22

81675 Munich, Germany

Tel.: +49 89 41405803, Fax.: +49 89 4140 4362

gregor.zimmermann@tum.de
